# Mechanical neuroscience: Emil du Bois-Reymond's innovations in theory and practice

**DOI:** 10.3389/fnsys.2015.00133

**Published:** 2015-09-30

**Authors:** Gabriel Finkelstein

**Affiliations:** Department of History, University of Colorado DenverDenver, CO, USA

**Keywords:** Emil du Bois-Reymond, neuroscience, paradigm, history, nineteenth-century

## Introduction

Contemporary neuroscience unites three scientific traditions: one in anatomy that goes back to the Greeks (functional localization), one in chemistry that arose in the twentieth-century (neurotransmitters), and one in physics that arose in the nineteenth-century (electrical signals) (Clarke and Jacyna, 1987; Finger, 1994; Valenstein, 2005; McComas, 2011). Emil du Bois-Reymond is the father of the last. He's not quite Victor Frankenstein, but he's close.

When du Bois-Reymond began his investigations neurophysiologists were divided. Some followed the physician Luigi Galvani (1737–1798) in ascribing the action of nerves and muscles to vital powers of “animal electricity.” Others sided with the physicist Alessandro Volta (1745–1827) in believing that the muscular contractions that Galvani observed were an artifact of electricity generated by the contact of metal with organic tissue (Pera, 1992; Piccolino, 1998; Piccolino and Bresadola, 2013). Recognizing value in both positions, du Bois-Reymond solved the problem of contact electricity, set forth a program of biological reduction, and demonstrated the electrical nature of nerve signals. In a little less than two years—from March, 1841 to January, 1843—he created the discipline of electrophysiology.

That's the short version of du Bois-Reymond's innovation. We know that it's true because winners write history—at least, that's what we've been told. In the case of du Bois-Reymond, however, the truth is closer to this: those who write history win. Does this fact change his story? Consider the evidence.

## Theory

Emil du Bois-Reymond was born in Berlin on 7 November 1818 to family of Huguenot origins (Finkelstein, 2013). His mother's forebears were merchants, scholars, and artists that had lived in Berlin for generations; his father was a poor, self-taught immigrant from the Swiss canton of Neuchâtel. Du Bois-Reymond grew up speaking French, reading the *philosophes*, and chafing at the confines of his father's Calvinism. His first impulse on entering university was to embrace Romanticism, but it didn't take long for him to ditch *Naturphilosophie* in favor of a mechanical view of the world. Clues to his conversion can be found in his reading: Lucretius in the summer of 1838, French physics shortly thereafter, and the *Cours de philosophie positive* of Auguste Comte—most likely—in 1842. The result, as he reported to a friend, regarded science from a perspective that was nearly Cartesian:

I have sworn to uphold the truth that no forces operate in the organism other than those common to physics and chemistry; that, where these do not suffice in explanation by means of the mathematico-physical method, one must either look at a specific case of the force in question, or one must assume new forces, which, of the same order as the physico-chemical inherent in matter, always reduce to only attractive and repulsive components (du Bois-Reymond, 1918, p. 108).

It should be noted that du Bois-Reymond's materialism was purely methodological. As he saw it, force and matter served as terms of convenience, and anyone who confused them with real entities was committing the same error as primitive ancestors who “once peopled bush and fountain, rock, air, and sea with creatures of their imagination” (du Bois-Reymond, 1848–1884, pp. xl–xli). Anthropomorphic fantasies had no place in science.

With respect to biology du Bois-Reymond's outlook translated into a repudiation of vitalism. To be sure, he was not the first German to adopt this position—the Romantic psychiatrist Johann Christian Reil (1759–1813) had done so in 1796, as had the psychologist Hermann Lotze (1817–1881) in 1842—but du Bois-Reymond was the first to effect a lasting change in the outlook of his colleagues. It was one thing for theoreticians like Reil and Lotze to espouse a particular view of nature, and it was altogether another for a scientist like du Bois-Reymond. This is why his words carried so much weight. Looking back at the development of his discipline over the course of the nineteenth-century, the Belgian physiologist Paul Heger (1846–1925) deemed du Bois-Reymond's pronouncement “an act of courage whose worth is difficult to appreciate today” (Heger, 1896–1897, p. 565).

In this regard the three elements of du Bois-Reymond's positivism (biological reduction, functional analysis, and methodological materialism) constitute a shift in the paradigm of neuroscience. Very few neuroscientists still endorse the crude materialism that Carl Vogt (1817–1895), Jacob Moleschott (1822–1893), and Ludwig Büchner (1824–1899) popularized in their polemics against religion in the 1850s (Gregory, 1977). By contrast, most share du Bois-Reymond's opinion that vital powers do not operate in biology, that explanations of the nervous system require accounts of the chemical and physical processes that drive it, and that certain problems, like the ultimate essence of matter, consciousness, and free will, lie beyond the understanding of science.

## Practice

**Figure 1:**
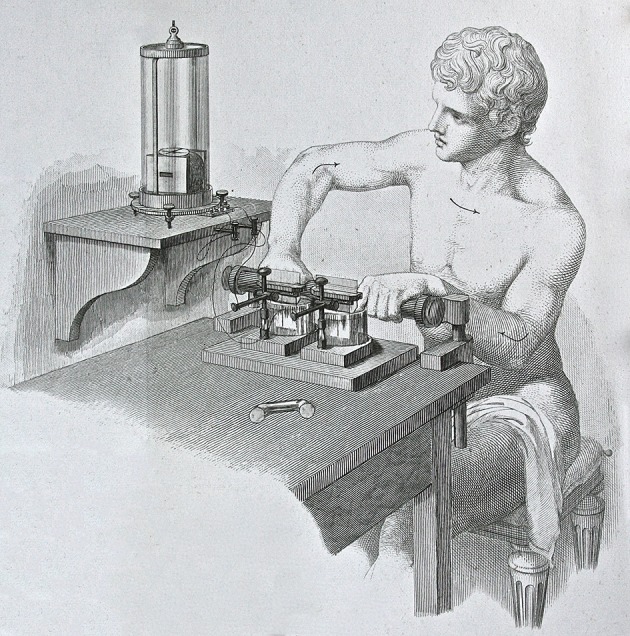
**Du Bois-Reymond's demonstration of voluntary tetanic current**. The arrows indicate the flow of current through the body. The galvanometer detecting the signal rests on a separate shelf to avoid disturbance. Reprinted from du Bois-Reymond (1848–1884), Volume 2, part 2, plate V, Figure 147.

Perhaps the greatest of du Bois-Reymond's innovations was an experimental design that solved three problems that vexed the study of animal electricity. First, du Bois-Reymond devised neutral means of coupling instruments to tissue, most notably “non-polarizable” electrodes formed from an amalgam of zinc, zinc sulfate, and modeling clay. Second, he invented devices like the “magneo-electrometer” (AC generator) and the “rheocord” (potentiometer) that delivered graded shocks to his preparations. Last, he constructed a galvanometer sensitive enough to record the results of his protocols. These breakthroughs allowed him to detect action currents in frog muscles in 1843; 4 years later the addition of a Wheatstone bridge circuit to his set-up let him to demonstrate the same electrical signals in human subjects (see Figure 1) (Finkelstein, 2013).

Du Bois-Reymond's most famous experiment proceeded as follows. He attached the leads of his galvanometer to platinum plates resting in “conducting vessels” filled with saline. After immersing his fingers in the electrolyte, he waited for the galvanometer needle to come to rest. All of a sudden, he tensed one of his arms. The needle jumped. Observers in Berlin, Paris, and London were astonished by his exhibition; indeed the performance, which was simple and striking, made his name as much as any of his scholarly publications. The point was not lost on du Bois-Reymond, who went on to devise a panoply of demonstration aids like “the twitch telegraph,” “the frog alarm,” “the frog pistol,” “the mirror multiplier,” and “the feather myograph,” many of which still feature in the collections of science museums today.

The significance of du Bois-Reymond's instruments escaped his French and English colleagues at first, but within a generation even du Bois-Reymond's skeptics adopted his methods. In fact, it wouldn't be that much of a stretch to describe the history of neuroscience in the image of du Bois-Reymond: a march of technological progress from the galvanometer to the optogenetic sensor, all originating in table-top experiments that he carried out with apparatus he built himself.

Du Bois-Reymond's instrumental methods embodied a positivist commitment to measurement that permanently changed the practice of science (Kuhn, 1961, p. 190). Lord Kelvin expressed his attitude perfectly: “I often say that when you can measure what you are speaking about, and express it in numbers, you know something about it; but when you cannot measure it, when you cannot express it in numbers, your knowledge is of a meager and unsatisfactory kind: it may be the beginning of knowledge, but you have scarcely in your thoughts advanced to the stage of *science*” (Thompson, 1889, p. 73). It might be argued that our knowledge of the brain remains meager and unsatisfactory, that there is still no consensus about what to measure, at what scale, and over what span of time. All the same, Alan Lloyd Hodgkin and Andrew Huxley's mathematical model of neuron action potentials endures as one of the great achievements of twentieth-century physiology. Had du Bois-Reymond been alive to hear the news of their Nobel Prize, he would have endorsed it implicitly.

## Communication

None of du Bois-Reymond's innovations would have had quite the same effect if he hadn't advertised them to colleagues, students, and the public. Fortunately, he possessed an exceptional talent at communication. He helped found the Berlin Society of Physics and the Berlin Society of Anthropology, organizations that served as sounding boards for his discoveries; he worked as permanent secretary of the Prussian Academy of Sciences, rector of the University of Berlin, and dean of the medical school, positions that required him to deliver formal addresses; and he edited the leading German journal of physiology, a post that helped him shape the discipline. He also directed Prussia's first institute of physiology, instructed medical students in animal electricity, and taught Berlin's most popular class. Finally, he conducted demonstrations in Berlin, Paris, and London, spoke to audiences throughout Germany and the Netherlands, and reprinted his speeches in the *Deutsche Rundschau*, the *Revue scientifique, Nature*, and the *Popular Science Monthly*.

Like all great performers du Bois-Reymond thought hard about his craft. He compared professors who spoke poorly to eccentrics who wandered about in their dressing-gowns (du Bois-Reymond, 1912, p. 492). Remarking on one public session at the Academy of Sciences, he told his wife, “The only decent address was mine, of course… profound, allusive, brief, masterfully arranged. Caviar for the audience” (du Bois-Reymond, 1882). He used the same idiom to describe the lectures of his tour of the Rhineland: “Apparently no one is aware that you can present a scientific discourse with a garnish of poetic reflections and numerical figures, like a filet with mixed pickles and olives. In Cologne they insisted that I had read from a manuscript” (du Bois-Reymond, 1877).

Du Bois-Reymond's attention to rhetoric was deliberate. Early in his career he realized that “popularizers of science persist in the public mind as memorial stones of human progress long after the waves of oblivion have surged over the originators of the soundest research” (du Bois-Reymond, 1912, p. 354). He never abandoned his investigations of animal electricity, but he also made certain to address his audience. “The time of poetic production in European nations appears to have passed,” he wrote to his fiancée in 1853, “and talent, which might otherwise have achieved something there, throws itself into oratory and journalism, and later dabbles in politics” (du Bois-Reymond, 1853). He was somewhat blunter about the change two decades later. “Today Goethe would be holding forth at the Reichstag” (du Bois-Reymond, 1912, p. 607).

Du Bois-Reymond's mention of Goethe alluded to his essay “The Epochs of Thought.” In this short work, Goethe divided history into four ages: Poetry, Theology, Philosophy, and Prose, the last of which he judged a disaster (Goethe, 1817). Du Bois-Reymond didn't entirely share Goethe's pessimism, perhaps because he equated the prosaic age with science, and perhaps because he felt more comfortable at the lectern. Whatever the reason, du Bois-Reymond approached writing with a self-awareness that verged on irony. He was acutely aware of his place in the history of science, and his accounts of it draw from the language of scripture and legend. In a literal sense, du Bois-Reymond not only shifted the paradigm of neuroscience, he also set it.

### Conflict of interest statement

The author declares that the research was conducted in the absence of any commercial or financial relationships that could be construed as a potential conflict of interest.

## References

[B1] Clarke E. Jacyna L. S. (1987). Nineteenth-Century Origins of Neuroscientific Concepts. Berkeley; Los Angeles; London: University of California Press.

[B2] du Bois-Reymond E. (1848–1884). Untersuchungen über thierische Electricität. Vol. 2 Berlin: Reimer.

[B3] du Bois-Reymond E. (1853). Letter to Jeannette du Bois-Reymond. Berlin: Staatsbibliothek zu Berlin, Preußischer Kulturbesitz, Haus Potsdamer Straße, Handschriftenabteilung.

[B4] du Bois-Reymond E. (1877). Letter to Jeannette du Bois-Reymond, Elberfeld: Staatsbibliothek zu Berlin, Preußischer Kulturbesitz, Haus Potsdamer Straße, Handschriftenabteilung.

[B5] du Bois-Reymond E. (1882). Letter to Jeannette du Bois-Reymond, Berlin: Staatsbibliothek zu Berlin, Preußischer Kulturbesitz, Haus Potsdamer Straße, Handschriftenabteilung.

[B6] du Bois-Reymond E. (ed.). (1912). Reden von Emil du Bois-Reymond. Leipzig: Veit.

[B7] du Bois-Reymond E. (ed.). (1918). Jugendbriefe von Emil du Bois-Reymond an Eduard Hallmann. Berlin: Reimer.

[B8] Finger S. (1994). Origins of Neuroscience: A History of Explorations into Brain Function. New York, NY: Oxford University Press.

[B9] Finkelstein G. (2013). Emil du Bois-Reymond: Neuroscience, Self, and Society in Nineteenth-Century Germany. Cambridge; London: The MIT Press.

[B10] Goethe J. W. (1817). Geistes-Epochen, nach Hermanns neuesten Mitteilungen. Über Kunst und Altherthum 1, 107–112.

[B11] Gregory F. (1977). Scientific Materialism in Nineteenth Century Germany. Dordrecht; Boston: Reidel.

[B12] Heger P. (1896–1897). Emil du Bois-Reymond. Conférence donnée à l'Association des Étudiants en médicine de l'Université libre de Bruxelles, le 26 mars 1897. Revue de l'Université de Bruxelles 2, 565.

[B13] Kuhn T. S. (1961). The function of measurement in modern physical science. Isis 52, 161–193. 10.1086/349468

[B14] McComas A. J. (2011). Galvani's Spark: The Story of the Nerve Impulse. New York, NY: Oxford University Press.

[B15] Pera M. (1992). The Ambiguous Frog: The Galvani-Volta Controversy on Animal Electricity, tr. Jonathan Mandelbaum. Princeton: Princeton University Press.

[B16] Piccolino M. Bresadola M. (2013). Shocking Frogs: Galvani, Volta and the Electric Origins of Neuroscience. Oxford: Oxford University Press.

[B17] Piccolino M. (1998). Animal electricity and the birth of electrophysiology: the legacy of Luigi Galvani. Brain Res. Bull. 46, 381–407. 10.1016/S0361-9230(98)00026-4 9739001

[B18] Thompson W. (1889). Electrical units of measurement. Popular Lect. Address. 1, 73–136.

[B19] Valenstein E. S. (2005). The War of the Soups and the Sparks: The Discovery of Neurotransmitters and the Dispute Over How Nerves Communicate. New York, NY: Columbia University Press.

